# Improving bereavement outcomes in Zimbabwe: protocol for a feasibility cluster trial of the 9-cell bereavement tool

**DOI:** 10.1186/s40814-019-0450-5

**Published:** 2019-05-10

**Authors:** Barbara Mutedzi, Lisa Langhaug, Jennifer Hunt, Kennedy Nkhoma, Richard Harding

**Affiliations:** 1 Island Hospice and Healthcare, 6 Natal Road, Belgravia, Harare, Zimbabwe; 2 Harare, Zimbabwe; 3 Florence Nightingale Faculty of Nursing, Midwifery and Palliative Care, Cicely Saunders Institute of Palliative Care, Policy and Rehabilitation, King’s College London, Bessemer Road, London, SE5 9PJ UK

**Keywords:** Bereavement, Grief, Bereavement tool, Feasibility cluster trial, Zimbabwe, Africa

## Abstract

**Background:**

The high burden of bereavement in sub-Saharan Africa is largely attributable to HIV, cancer, and other non-communicable diseases. However, interventions to improve grief and bereavement are rare. Given high rates of mortality in the context of weak health systems, community lay members are well placed to provide peer bereavement support. The 9-cell bereavement tool was developed in Zimbabwe to improve community members’ capacity to support the bereaved. This study aims to determine the feasibility of implementing the 9-cell bereavement tool and recruitment to experimental evaluation.

**Methods/design:**

This feasibility cluster randomized trial with embedded qualitative interviews will be conducted in two comparable neighborhoods in Zimbabwe. Community leaders from each neighborhood will identify 25 potential community lay bereavement supporters, each of whom will recruit 2–3 bereaved community members into the trial. The intervention will be randomly allocated to one community, and the second community will form a wait-list control (*n* ≥ 75 in each community cluster). Recruitment is estimated to take place over 3 weeks. Measures at T0 (baseline, i.e., week 0), T1 (midline, i.e., week 14 or 3 months post-baseline) and T2 (endline, i.e., week 27 or 3 months post-midline) will address mental health, social support, and levels of grief per individual. Qualitative data will describe lay supporters’ views of intervention training and delivery, and participants’ experience of bereavement support.

**Discussion:**

This is the first documented trial evaluating a bereavement intervention in sub-Saharan Africa. Recruitment, retention, and measurement data will determine the feasibility of a full trial.

**Trial registration:**

ISRCTN, ISRCTN16484746. Registered 6 February 2018

## Background

Bereavement is the process during which grief is experienced over time [1]. Both grief and bereavement are subjective in their experience and duration. Bereaved individuals who have not been through the process of grief have an increased risk of mortality [2, 3], deterioration of physical health [2], reduced cognitive functioning, increase in mental health challenges, and associated illnesses [4]. These outcomes negatively impact the socio-economic status of individuals while generating high costs in already fragile economies of low-income countries [5, 6]. Bereavement support is therefore an essential and core component of palliative care defined by the WHO [7, 8].

Bereavement is an under-researched field in African countries, despite high mortality rates from communicable diseases such as HIV, tuberculosis, and malaria; and non-communicable diseases such as cancer, heart disease, suicide, and sudden deaths [9, 10]. The WHO Global Palliative Care Atlas, the Lancet Commission on Pain and Palliative Care [11], and the World Health Resolution on Palliative Care [12] have all identified a critical gap between the need for, and provision of, palliative care (including bereavement care). However, within this gap, there is further inequity. Systematic reviews of the evidence in sub-Saharan Africa have found that bereavement interventions are rarely described within palliative care intervention studies [13, 14].

Community-based interventions using already existing structures, for example, local health cadres embedded in the local health system, are more effective and widely accepted within low-resourced countries such as Zimbabwe, whose socio-economic structures have vastly deteriorated [15]. Previous studies have shown that local health cadres trained to act as an extension of the central health centers are effective in health delivery as they increase coverage and access [16]. Caregivers from the communities that local health services serve can offer in-depth knowledge of local cultural preferences and practices for effective delivery and uptake of healthcare services [17–19].

This study will adopt the 9-cell bereavement intervention developed in Zimbabwe. The 9-cell was designed to assist individuals to reflect on their feelings in bereavement and identify resources in families and communities to manage bereavement. This process is intended to increase the lay supporter’s understanding of the experience of grief and to identify ways of increasing support to the bereaved. The 9-cell bereavement tool explores the communication that the bereaved currently receive, discussing and linking their own grief and bereavement experiences with the support they need at different stages [20]. This person-centered approach is designed to provide context-based, culturally appropriate, individually tailored support. The tool was implemented in Tanzania and India, with process data suggesting increases in awareness of the concepts behind grief and the bereavement process, providing rare opportunities to share experiences of both with fellow community members. One year after the 2004 Tsunami, an NGO funded a 10-week training and supervision program to integrate the 9-cell bereavement tool into the work of volunteer counselors in Tamil Nadu, India. Despite these examples of use, there has been no rigorous evaluation of the 9-cell [21].

There is an absence of effectiveness literature for bereavement interventions in sub-Saharan Africa [22–24]. However, the positive outcomes experienced in the few documented interventions, including the 9-cell bereavement tool as tested in other low-income countries similar to Zimbabwe, support the need for community-based bereavement interventions to be scaled up. With this knowledge and due to the potential vulnerability of bereaved individuals, the scarcity of resources within sub-Saharan Africa, it is essential to therefore establish the feasibility of delivering and testing the tool using a trial design, prior to investment and participant involvement in a full clinical trial.

## Methods

### Aim and objectives

The aims of this study are to assess the feasibility of implementing the 9-cell bereavement tool at the community level and to determine the feasibility of evaluating the intervention using a cluster randomized control trial design.

The objectives are (i) to determine the feasibility of conducting a randomized cluster trial in terms of recruitment and retention; (ii) to assess the feasibility of implementing the 9-cell bereavement tool; (iii) to determine whether there would be contamination between the clusters; (iv) to assess the acceptability and completeness of measures and data; (v) to identify trial participants’ views and experience of the intervention and its mechanisms of action; (vi) to estimate potential effect size; and (vii) to determine whether a full trial is warranted.

### Feasibility questions and success criteria

We will seek to answer the following research questions: (1) will we be able to recruit at least 75% of the suggested sample size of interventionists and trial participants interventionists within 3 weeks? In order to meet proposed trial timelines, we suggest trial recruitment criteria of recruiting community leaders within 1 week; interventionists within 1 week and the trial participants within 1 week; giving a total recruitment period of 3 weeks. (2) Will we be able to retain at least 75% of both the trial participants and the interventionists in the total duration of the study? (3) Can we deliver the 9-cell bereavement intervention as intended? (4) Will the processes of a randomized cluster trial be possible? (5) Does process data suggest that the anticipated effect is likely?

There is a paucity of guidance on setting progression criteria for feasibility and pilot trials [25]. Therefore, we draw on the MRC guidance that such criteria should be judged in light of all study findings and used to refine study design. Our recruitment and retention criterion of 75% was set in light of published feasibility trial criteria and reflects the nature of our population (i.e., they are community-dwelling bereaved individuals without any known serious health conditions and so we anticipate high retention).

## Study design

A cluster feasibility randomized control trial with nested qualitative focus groups will be used for this study. Cluster randomization would be required in a full trial as the intervention is delivered at the community level. Recruitment is estimated to take place over an estimated 3-week period. It will include approaching and sensitization of community leaders; identification and recruitment of interventionists; qualitative data collection (FGD 1) with recruited interventionists; and identification and recruitment of trial participants.

**Fig. 1:**
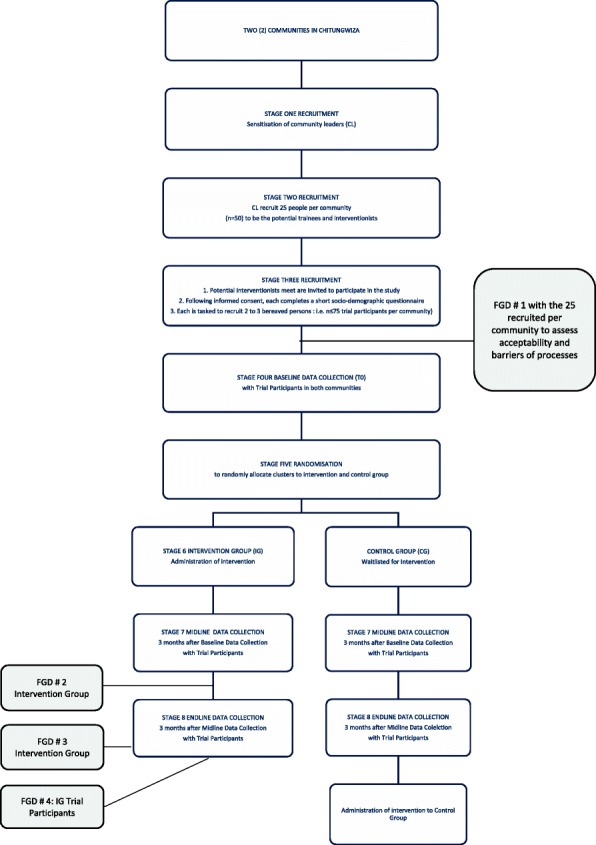
Study flow chart

The data collection timeline as illustrated in Fig. 1 will be as follows:

**Table:** 

Week 0	Baseline data collection from trial participants
	Randomization
	Administering intervention to the intervention group
Week 14	Midline data collection from trial participants
	Qualitative data collection (FDG 2) with the intervention group
Week 27	Endline data collection from trial participants
	Qualitative data collection (FDG 3) with the intervention group
	Qualitative data collection (FGD 4) from trial participants
	Administering intervention to control group

Figure 1 presents the study flow chart. This is followed by a narrative description of the data collection stages.

### Timeline

Interventions are more effective when administered closer (though not too soon) to the time of death of the loved one [26, 27]. Six to 18 months is a suitable period for administering bereavement interventions *inclusive* of their follow-up [26]. According to Jennifer Hunt, the developer of the 9-cell bereavement tool, the 6-month mark usually represents a time when social support often fades and the reality of the loss sets in. Inclusion criteria (elaborated in the “Recruitment” section below) of *trial participants* will therefore be individuals in the community who have experienced the loss of a loved one in the past 6 months from the recruitment date (even where someone has been bereaved within a week of the study; by the time the study is finished it will be over 9 months, so they theoretically are still eligible as 6 to 18 months is a suitable period for administering interventions inclusive of their follow-up as indicated above).

### Setting

This study will be conducted in Chitungwiza, a city bordering the capital city of Zimbabwe. A local supporting organization for this study, Island Hospice and Healthcare (IHH), has been involved in community work in Chitungwiza for the past 15 years engaging community groups, community leaders, non-governmental organizations (NGOs), churches, and government ministries.

**Fig. 2:**
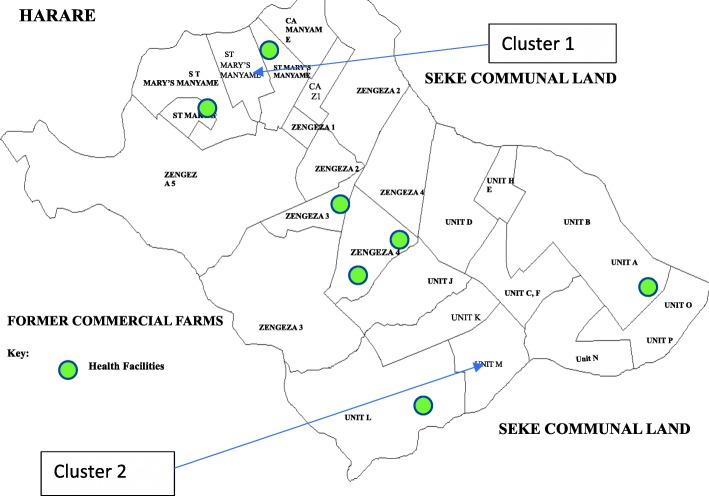
Map of Chitungwiza

Two broadly similar communities within Chitungwiza have been selected; one will act as the intervention group (IG) and the other as the control group (CG). Chitungwiza is densely populated with a large surface area, which greatly reduces potential contamination between the two selected intervention and control areas. Figure 2 provides the map of Chitungwiza; one community cluster is in the north-west section and the second is in the south-east, therefore reducing the likelihood of contamination. They have similar socio-demographic characteristics and value systems, with the economy being largely supported by informal trade and falling under the same traditional territory and health administration. The two communities selected within Chitungwiza are about 8 km apart. Through discussion with local stakeholders, 8 km is anticipated to be adequate distance and corresponding density to reduce possible contamination between the two clusters. However, as an added precaution, the control group will be asked at midline and at endline to establish whether they have visited the community where the intervention took place and if they had a discussion with any community member who received the intervention.

A local community hall or other forms of available and convenient gathering space will be identified in the respective communities as central data collection venues. The community leaders assisting in the recruitment processes will assist in identifying central venues convenient to participants recruited for the interventions and the study. Recruitment processes are detailed in the following section.

### Sample size

Past research has recommended sample sizes of 24 and 50 [28–31]. For this feasibility study, sample sizes for community leaders have been set at 25 in total, and for interventionists, at 25 per cluster; with the trial participants set at 75. The target is 50; however, a reach of 75 has been set, to allow for any challenges in recruitment and for possible attrition from the study.

### Recruitment

The recruitment process follows three stages.

#### Stage 1: Recruitment of community leaders

The recruitment process will begin with study engagement using existing relationships that Island Hospice and Healthcare (IHH) has with community leaders (CL) from the selected two communities in Chitungwiza. The rationale, goals, and intended procedures of the study will be explained, community leader permission sought to undertake the research, and support from the community leaders (CL) requested to deliver the study. This is an essential step in conducting community-based research in this context.

#### Stage 2: Recruitment of potential interventionists by community leaders

The community leaders (CL) will be tasked to invite 25 people (i.e., potential interventionists) from their respective communities to attend a meeting. Inclusion criteria for potential interventionists will include individuals from the two selected communities of Chitungwiza which the CL serve; recruited by the community leaders; be aged at least 18 years of age; be able to either verbally consent or be able to consent in writing; be able to read and write; and expected to be able to deliver the lay community peer intervention. At the meeting, the potential interventionists will be provided with information about the study and asked to give informed consent, to participate in the study. Interventionists will complete a short questionnaire that will assess their socio-demographic background and bereavement history. The questionnaire is self-administered. The researchers will provide instruction to the interventionists and be available to assist with clarification of any questions where required. The third activity is a focus group discussion (FGD#1 as illustrated in Fig. 1) with the interventionists, to assess the feasibility of them identifying potential trial participants and inviting them to meet with the researchers, to learn more about the study.

#### Stage 3: Recruitment of potential trial participants

The potential interventionists from each community will be tasked to identify 2 to 3 people according to the following criteria. Potential participants must be (a) at least 18 years old, (b) resident within their neighborhoods, (c) someone with whom they interact with on a daily basis, (d) whom they know to have been bereaved in the past 6 months, (e) would have the ability to either verbally consent or be able to consent in writing, (f) be able to read and write, and (g) be expected to attend and participate in the study. Potential participants who meet all of these criteria will be invited to a meeting where information regarding the study will be shared. Researchers will obtain consent from potential trial participants.

Following written informed consent, potential trial participants will be invited to participate in the study. Participating in the study includes completing questionnaires at baseline (T0, i.e., week 0), midline (T1, i.e., 3 months post-baseline or week 14), and endline (T2, i.e., 3 months post-midline or at week 27), with a subsample of the intervention cluster participants invited to qualitative focus group interviews to better understand participants’ views on study participation and intervention mechanisms of action. Focus group participants will be aged at least 18 years of age; be able to either verbally consent or be able to consent in writing; and be able to verbally share their experiences and thoughts regarding the study.

#### Retention of interventionists and trial participants

Registration forms will include full names and contact details of both interventionists and trial participants. Contact details will be used to notify both interventionists and trial participants, of the planned data collection dates respectively. For example, mobile phone messages will be sent to all both the interventionists and the trial participants, requesting and reminding them of the dates for baseline, midline, and endline data collection.

#### Stage 4: Baseline data collection from trial participants

Once recruited into the study, the trial participants (*n* = 25 per community) will complete self-report questionnaires, both demographic and validated outcome measures. Data collection will be on different days for each community, but within a space of a week. Questionnaires will be self-administered, with assistance from researchers where needed. Trained surveyors will go through some sample questions with the respondents in order to familiarize them with the questionnaire format. The surveyors will be present to assist respondents who may require further assistance beyond initial instructions. Completing the questionnaires is estimated to take less than an hour.

The questionnaires used were selected as they are in line with the intended intervention outcomes, have been successfully tested to be culturally specific adaptation, have been used by other researchers in Africa and or other low- and middle-income countries, and their ease of understanding and therefore administration.

These are the Shona Symptom Questionnaire (SSQ, measure mental health), the Modified Social Support Survey (MSSS, measures social support), and the Texas Revised Inventory of Grief (TRIG, measures levels of grief). The SSQ was developed in Zimbabwe. It incorporates 14 questions, 7 of which are etic (global concept) and 7 are emic (culturally specific). The MSSS has 20 questions based on 4 subscales of emotional/informational, tangible, affectionate, and positive social interactions [32]. The TRIG is a 13-item, self-report measure of current feelings related to the loss [33]. As an illustration of their successful adaptability in different and varied cultural settings and languages, the MSSS and the TRIG have both been successfully used in both low- and high-income countries including Brazil—Portuguese language [34]; the USA [35]; in French Canada [36]; in New Mexico [37]; Norway [38]; and Spain [39].

#### Stage 5: Randomization

Random allocation will be conducted by a statistician independent of the study, who will use an electronic system to randomly allocate the two clusters to intervention (IG) and control group (CG). Both the researchers (except the principal investigator) and the data analyst will not be aware of which cluster will be the intervention group and which one will be the control group. This *blinding* reduces the influence that researchers may have when collecting the quantitative datasets from the community clusters [40] and any influence that the data analyst will have when analyzing the same data sets. The 25 potential interventionists in the allocated intervention cluster will receive the training and the 9-cell bereavement tool, as explained in Stage 6 below. The control group will be waitlisted to receive the intervention at the end of the study (following final endline data collection point).

#### Stage 6: Intervention

The 25 lay community members in the cluster randomized to be the intervention group (IG) will take part in the intervention of the 9-cell bereavement tool. The ex-bereavement service coordinator and trainer, responsible for the development of the 9-cell bereavement tool, will administer the 9-cell bereavement tool with assistance and translation from one Island Hospice Healthcare (IHH) social worker. Before the intervention, the trainer will provide intensive pre-intervention preparation with the IHH social worker in order to transfer skills to IHH as a secondary benefit of the study. IHH will support the administration of the 9-cell tool in the identified communities.

The 9-cell bereavement tool is administered in a 7-h (1 day) session. Community lay caregivers are an appropriate target as social support can improve grief and bereavement outcomes [41, 42].

It assesses personal feelings in relation to bereavement, identifies judgmental attitudes, inappropriate religious tenets, lack of understanding, effects of family, and community support. The 9-cell bereavement tool places the uniquely personal journey of grief firmly within the social setting in which grief is processed; how to ensure a better fit for the grieving individual within their society; is particularly useful in multicultural settings; and was developed from experience from local bereavement beliefs and practices.

The overall process of implementing the 9-cell bereavement tool:

- Builds on people’s existing knowledge and experience
- Models an open-minded and non-judgmental approach
- Recognizes great diversity in grief experiences in individuals, genders, families, cultures, and faiths
- Encourages intervention participants to listen to others while helping them to break down barriers of previously held beliefs about grief

Transport fees, incentives, and refreshments will be provided during the intervention as in the data collection phase of the study.

#### Stage 7: Midline data collection

At T1 (at week 14 of 3 months following baseline), all participants from both clusters will be invited to complete the questionnaires listed above. Data collection for the intervention group and the control group will be held on different days. Through discussion with local stakeholders, 8 km was anticipated to be adequate distance and corresponding density to reduce possible contamination between the two clusters. However, as an added precaution, the control group will be asked at midline and at endline to establish whether they have visited the community where the intervention took place and if they had a discussion with any community member who received the intervention.

Immediately after T1 midline data collection with trial participants, interventionists will be invited back to participate in a second focus group discussion (FGD#2 as identified in the study flow chart in Fig. 1). The semi-structured discussion guides with open-ended questions allow for in-depth explanations and descriptions of subjective experiences [43]. Open-ended questions encourage focus group participants to share their lived experiences without the constraints of structured tools [43]. The purpose of this second focus group discussion would be to appraise the intervention training received 3 months prior, and their ability to deliver the intervention in line with the information provided. Questions will be centered around their experience of their own bereavement process following the intervention, how they delivered the intervention, when they started to feel changes within themselves around the intervention concepts, the language they started using with others going through the same bereavement process, any changes in the way they interacted with others around bereavement, and the support they feel they would need currently and in the future, related to the intervention elements learned. FGD will take up to 45 min (see procedures below). The group discussions will be led by the principal investigator (BM) supported by a research assistant who will take notes. The discussions will be held in the respective communities, with refreshments and incentives provided as appreciative tokens for the respondents’ participation. Focus group discussions will be recorded with the participants’ permission (included in the informed consent form), and deleted once transcribed verbatim.

#### Stage 8: Endline data collection (T2)

At T2 (endline data collection; week 27 or 3 months post-midline), all trial participants will complete the questionnaires following procedures for the quantitative data collection described above. An additional data collection process will occur with the intervention group (IG). As they complete the final endline questionnaire, 10 to 15 participants will be purposively recruited into a focus group (FGD#4 as indicated in Fig. 1—the study flow chart). Purposive sampling will guide in recruiting at least an equal number per gender and age. In the group discussion, the principal investigator will share information about the intervention to the intervention group trial participants. Following the explanation, the trial participants, will join in a discussion centered on why they agreed to be in the study, what it was like for them, how often they saw the interventionist who recruited them into the study, what they discussed regarding bereavement, what they learnt to do or feel differently following the discussions, who initiated the discussions they had with the interventionists, any negative and or positive thoughts or actions that followed, discussions with others outside of the intervention, how the study helped, and if they felt they needed extra support they may require following the end of the study.

Once baseline, midline, and endline data collection, including the focus group discussions (FGD 1, 2, and 4, as indicated in the study flow chart in Fig. 1), a separate focus group discussion (FGD # 3) will be held with interventionists to assess their experience in interacting with the bereaved within their communities. This topic guide will ask questions around how they maintained the conversations with regard to bereavement with those in their communities, was it sustainable, were there any changes they noticed from the community in their renewed communication, do people actively seek them out, are they sort after, what do people want to discuss when they see them, are there self-described gains and if so what are they, will they use any of the lessons learned going forward, what will they use, what do they need to sustain this, and does anyone outside the trial ask for support from them?

## Data management

### Confidentiality

All completed consent forms will be kept in a locked room and cupboard at IHH, the local supporting organization. Only recognized research team members will have access. All analysis will be performed and saved on a password-protected laptop. No names will be included on the data collected; code/questionnaire numbers will have code numbers allocated to each respondent/participant’s responses. Questionnaires will be stored separately from the information and consent forms. Only the questionnaires will have the study ID numbers, not the information and consent forms. This further reduces any cross analysis.

### Compensation

All participants will be reimbursed for public transport to the study sites and will be provided with light refreshments at the venue. Participants will receive a small token of appreciation, valued at approximately USD3, to compensate them for the time taken to participate in the study. For their convenience, data collection will take place within the respective communities where the participants reside. Transport fare of an equivalent of USD2 will be provided to each participant.

### Risks

While it is believed that no harm can occur during this research, risks may include challenges in processing grief, high emotional burden, or distress. The 9-cell bereavement tool developer (JH) and a trained social worker from Island Hospice and Healthcare will be available during the sessions to assist where needed as they will be administering the intervention and are trained in counseling and working with the bereaved and highly distressed individuals. In addition, and as this project is supported by Island Hospice and Healthcare, participants can be referred for further counseling and assistance throughout the intervention period and following the evaluation.

## Data monitoring

Data monitoring (recruitment, retention, adverse events) will be reported to the Steering Committee meetings (chaired by the Chief Investigator, Richard Harding) by the local principal investigator (Barbara Mutedzi) for review.

## Data analysis

Quantitative data will be entered into a study-specific excel spreadsheet prior to import to SPSS for analysis.

In terms of recruitment, we will calculate the proportion of the target sample achieved for community lay interventionists and trial participants.

With respect to retention, we will calculate the proportion of potential trainees recruited who attended the full day training, and the proportion who remained active in bereavement support until trial end. We will report the proportion of trial participants who participate in T0 (baseline of week 0), T1 (midline at week 14 or 3 months post-baseline), and T2 (at week 27 or 3 months post-midline) data collection.

In terms of the outcome data, we will measure data completeness at each timepoint. We will plot data to check for floor and ceiling effects (to help us inform primary outcome selection), report data completeness, and plot means/standard deviations or medians and IQR (depending on data normality/symmetry of distribution) for each timepoint. We will appraise the selected outcome measures in terms of the qualitative explanation of perceived intervention benefits (and potential unintended consequences).

We will estimate potential treatment effect size using longitudinal methods for non-parametric data (no interim analysis), using an appropriate method (e.g., ANCOVA adjusting for baseline scores, with 95% confidence intervals). With regard to contamination, if we find that 8 km was a sufficient distance to minimize contamination, then we will (in full trial development) determine clusters that meet this criterion, which appears possible within Zimbabwe.

Verbatim transcripts of the individual and focus group interview data will be imported into NVIVO for analysis using the framework approach [44]. The trial participant and interventionist datasets will be analyzed separately. The thematic analysis will populate the framework by case analysis to generate themes, with the matrix permitting between-case comparison. Data will be integrated by appraising the quantitative outcomes in light of the qualitative description, with parity between the data types.

We will measure success in terms of our a priori criteria stated above, and if necessary refine methods by integrating the qualitative and quantitative data. If success criteria have not been met, refinements will aim to improve the design.

## Discussion

This study will assess the feasibility of implementing the 9-cell bereavement tool at a community level and to determine the feasibility of evaluating the intervention using a cluster randomized control trial design. This will enable us to determine optimal successful design for a full trial in an area that is currently neglected in the literature but has an enormous potential public health benefit. This protocol will enable us to provide a transparent analysis of the feasibility trial and reduce reporting bias [45]. We will appraise the results against the stated success criteria.

To reduce reporting bias, findings will be submitted for publication in peer-reviewed journals and be shared through the ethics review committees for wider distribution. Study results will guide a national bereavement strategy, promoting intervention dissemination across communities in Zimbabwe. Positive results from this study would support intervention rollout to many other African countries and training related to the feasibility study, and its methods will be shared regionally with other researchers in the same program.

## Ethics

Ethical approval was obtained from King’s College London (17/18-5415) and from the Medical Research Council of Zimbabwe (MRCZ) reference number MRCZ/A/2230.

## Abbreviations

AIDS: Acquired immunodeficiency syndrome
APCA: African Palliative Care Association
BuildCARE: Building Capacity, Access, Rights and Empowerment
CG: Control group
CL: Community leaders
FGD: Focus group discussion
HIV: Human immunodeficiency virus
HOSPAZ: Hospice and Palliative Care Association of Zimbabwe
HPCA: Hospice and Palliative Care Association
IG: Intervention group
IHH: Island Hospice and Healthcare
KCL: Kings College London
MOSSSS: Medical Outcomes Study Social Support Survey
MRCZ: Medical Research Council of Zimbabwe
NGO: Non-governmental organization
PI: Principal investigator
RCT: Randomized control trial
REFS: Resolution on palliative care
SPSS: Statistical Package for the Social Science
SSQ: Shona Symptom Questionnaire
TB: Tuberculosis
TRIG: Texas Revised Inventory Grief
UK: United Kingdom
USD: United States (American) Dollar
WHO: World Health Organization
ZDHS: Zimbabwe Demographic Health Survey
